# Introductory Note

**Published:** 1889

**Authors:** Edmund J. Lee

**Affiliations:** Philadelphia


### INTRODUCTORY NOTE.

THE editor has been collecting and arranging material for this repertory for more than ten years, yet he does not consider his results by any means complete. His sole endeavor has been to edit a new repertory which should be accurate and reliable, and at the same time as complete as he could make it. The material used has been collected from all reliable sources. After the death of Dr. Constantine Lippe, all the MSS. he had written for the second edition of his repertory was secured, and is included in this work. This repertory might, in fact, be considered as the second edition of Dr. Lippe's book, with such additions and corrections as the present editor has made.

The works of Hahnemann, Bœnninghausen, Hering, Lippe, Jahr, Dunham, etc., have been used. Besides these, the editor has had valuable aid from Dr. Edward Rushmore, who has sent him over three hundred pages of notes; also from Dr. S. A. Kimball, who collected many valuable notes from the interleaved repertory of Dr. W. P. Wesselhœft, and carefully revised the chapter on "Desires and Aversions;" from Dr. J. T. Kent's interleaved repertory many very valuable symptoms have been gathered. In the past ten years the editor has received numberless and most valuable hints from the late Dr. Ad. Lippe; the celebrated repertory of Bœnninghausen has been translated especially for this work; from Dr. E. W. Berridge notes and corrections have been received, and have been of great value. Drs. Walter M. James and George H. Clark will assist the editor in proof-reading, and in other ways lend valued assistance.

The chapter on "Mind and Disposition" includes especially the works of Hering and Jahr on this subject.

The editor would be very glad to receive notice of all errors detected.

EDMUND J. LEE.

PHILADELPHIA, April, 1889.

